# Self-Rated Symptoms of Oppositional Defiant Disorder and Conduct Disorder: Factor Structure, Reliability, and Validity in a Clinical Sample of Adolescents

**DOI:** 10.1007/s10578-024-01802-2

**Published:** 2024-12-11

**Authors:** Simon Klos, Ann-Kathrin Thöne, Manfred Döpfner, Anja Görtz-Dorten

**Affiliations:** 1 Faculty of Medicine and University Hospital Cologne, School for Child and Adolescent Cognitive Behavior Therapy (AKiP), University of Cologne, Pohligstr. 9, 50969 Cologne, Germany; 2 Faculty of Medicine and University Hospital Cologne, Department of Child and Adolescent Psychiatry, Psychosomatics and Psychotherapy, University of Cologne, Robert-Koch-Str. 10, 50931 Cologne, Germany

**Keywords:** Self-ratings, Oppositional defiant disorder [ODD], Conduct disorder [CD], Psychometric properties, Factor analysis

## Abstract

This study examined the psychometric properties of self-rated symptoms of oppositional defiant disorder (ODD) and conduct disorder (CD). We analyzed symptom ratings from a clinical outpatient sample of 658 adolescents aged 11;0–17;11 years diagnosed with ODD/CD and/or attention-deficit hyperactivity disorder. In two steps, we examined the factor structure of ODD models and an additional CD symptom dimension using confirmatory factor analysis (CFA) and exploratory structural equation modeling (ESEM). Our preferred three-factor CFA and ESEM models (CFI = 0.937–0.975, TLI = 0.926–0.962, RMSEA = 0.049–0.068, SRMR = 0.045–0.070) demonstrated scalar measurement invariance across age groups (≥ 14;0 years) and metric measurement invariance across informants (parents, teachers). With exceptions, the self-rated symptom scales showed acceptable internal consistencies (α = 0.68–0.84). Convergent (*r* =.27–.65) and divergent validity (*r* =.13–.41) were supported using Achenbach’s Youth Self Report Form. Our results provide implications for using self-rated ODD/CD symptoms in clinical practice and research.

**Supplementary Information:**

The online version contains supplementary material available at 10.1007/s10578-024-01802-2.

Oppositional defiant disorder (ODD) and conduct disorder (CD) are common mental disorders in children and adolescents (worldwide prevalence: 5.7%, 95% confidence interval: 4.0–8.1) [1]. Both disorders are frequent reasons for referral to healthcare services [2] and are associated with negative long-term outcomes in terms of mental and physical health, economic problems, and engaging in violence towards others [3].

To validly assess ODD and CD symptoms, it is necessary to obtain information from different informants (e.g., parents, teachers, youth) and using multiple methods (e.g., questionnaires, interviews, observations) [4, 5]. Comprehensive rating scales are an important part of the assessment process [6], and encompass empirically based rating scales [7] and symptom ratings based on diagnostic criteria from the *Diagnostic and Statistical Manual of Mental Disorders* (DSM-5) [8] or the *International Classification of Diseases and Related Health Problems* (ICD-10) [9]. Corresponding symptom ratings have proven to constitute a valid method to assess ODD and CD symptoms in children and adolescents, based on parent ratings [10, 11], teacher ratings [12], semi-structured clinical interviews [13], and self-ratings [14].

While parents and teachers are often mentioned as useful informants, caution is recommended when integrating self-ratings from children and adolescents, with an underreporting of conduct problems being a typical concern [15, 16]. Moreover, research in community and clinical samples has revealed that self-ratings have limited additional value for detecting externalizing mental disorders [15]. By contrast, other evidence suggests that self-ratings can provide valuable diagnostic information [5, 17–19], and research in community samples has even demonstrated that children and adolescents report more externalizing behavior problems compared to their parents [20]. Additionally, self-ratings are an important part of research on the stability of externalizing psychopathology [3, 21]. However, research about the psychometric properties of self-rated ODD/CD symptoms is rare, and greater clarity is needed in order to include self-ratings in clinical practice and further research.

According to the DSM-5, ODD can be defined as a pattern of *angry/irritable mood*, *argumentative/defiant behavior*, or *vindictiveness* lasting for at least six months [8]. All eight ODD symptoms listed in the DSM-5 receive equal weight with respect to diagnosing ODD. As such, the DSM-5 model can be characterized as a unidimensional model, in which all ODD symptoms load onto one common overarching ODD dimension. However, previous research has indicated that several dimensions can be identified within the ODD symptomatology [22]. Although the various models differ in terms of number and naming of the dimensions, they share the postulation of a more affective dimension (e.g., irritability) and a more behavioral dimension (e.g., defiant/headstrong), which are highly correlated with one another [23]. Well-known two-factor ODD models have been postulated by Burke and Loeber [24] and Rowe et al. [25], while three-dimensional models have been postulated by Stringaris and Goodman [26], Aebi et al. [27], and Burke et al. [28] (see Fig. 1). Due to the lack of consensus regarding which symptoms belong to which dimensions, previous studies used confirmatory factor analysis (CFA) to test various ODD models against each other [29–35], which resulted in further ODD models [35]. Previous research on the factor structure of ODD symptoms is almost exclusively based on teacher and parent ratings. To the best of our knowledge, only two studies have compared different ODD models based on adolescents’ self-ratings, and either only compared a unidimensional versus a multidimensional ODD model [21], or compared three ODD models with each other using the Youth Self Report (YSR) [36]. Moreover, as both of these studies were conducted in community samples, it remains unclear to what extent their findings may be generalized to the clinical population.

**Fig. 1:**
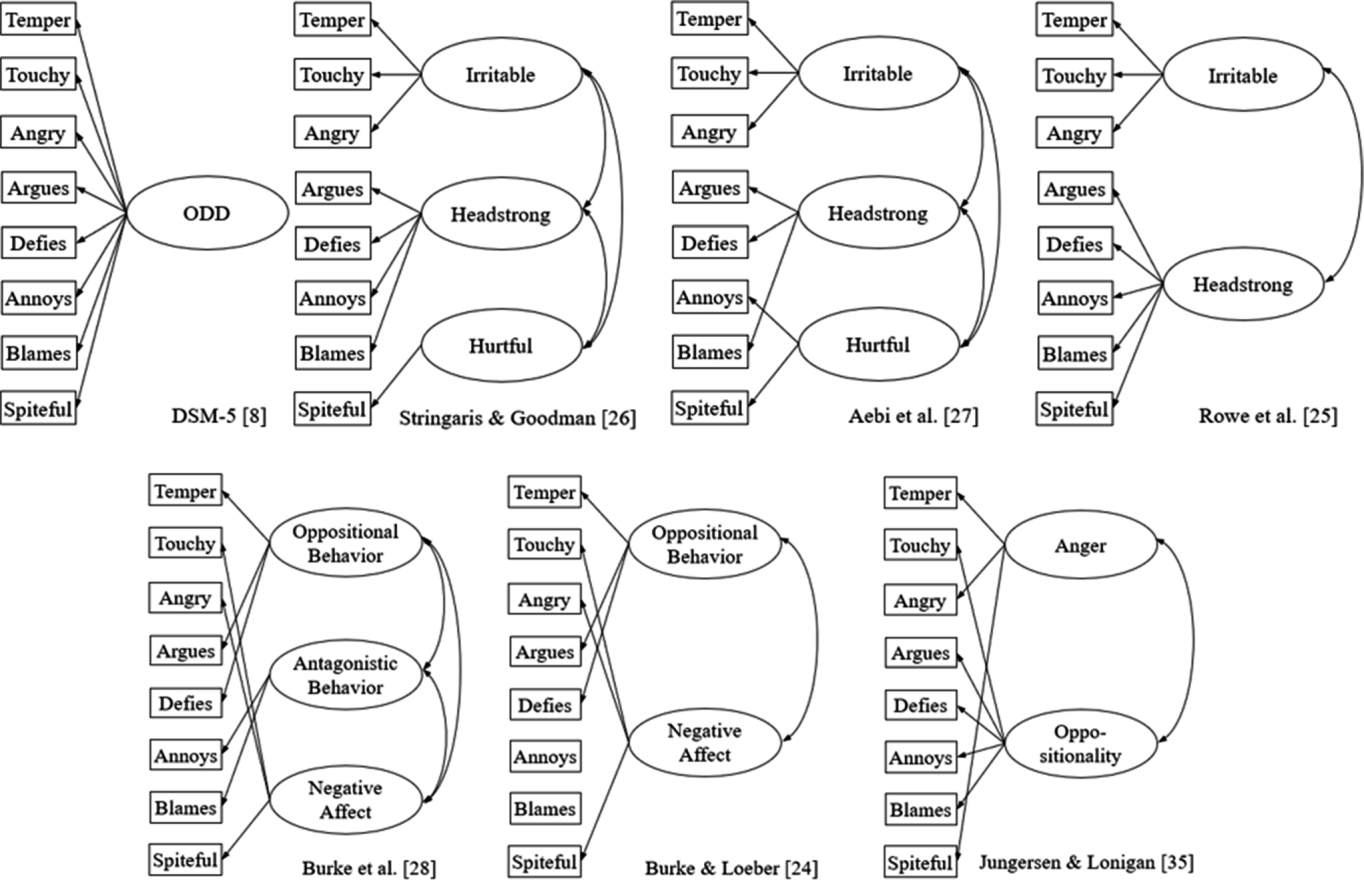
Overview of Existing ODD Factor Models

**Table 1:** Goodness-of-fit indices and Information Criteria of ODD Factor Models

Models	χ^2^ (df)	CFI	TLI	RMSEA (90% CI)	SRMR	AIC ^a^	BIC ^a^
Unidimensional model [8]	338.317* (20)	0.923	0.892	0.156 (0.141, 0.170)	0.062	12392.881	12500.622
Two-factor models							
Burke & Loeber [24]	197.719* (8)	0.948	0.902	0.190 (0.167, 0.213)	0.054	9417.174	9502.468
Rowe et al. [25]	139.916* (19)	0.971	0.957	0.098 (0.083, 0.114)	0.041	12236.667	12348.897
Jungersen & Lonigan [35]	326.935* (19)	0.926	0.890	0.157 (0.142, 0.172)	0.061	12389.583	12501.813
Three-factor models							
Aebi et al. [27]	97.349* (17)	0.981	0.968	0.085 (0.069, 0.101)	0.035	12213.343	12334.552
Burke et al. [28]	272.311* (17)	0.938	0.898	0.151 (0.136, 0.167)	0.054	12324.465	12445.674

*Note. N* = 658 self-ratings. χ^2^  = chi-square test of model fit; *df* = degrees of freedom; CFI = comparative fit index; TLI = Tucker-Lewis index; RMSEA = root mean square error of approximation; CI = confidence interval; SRMR = standardized root mean square residual; AIC = Akaike information criterion; BIC = Bayesian information criterion. ^a^ Calculated using maximum likelihood estimation with robust standard errors (MLR) for continuous indicators. *p <.01

**Table 2:** Goodness-of-fit indices and Information Criteria of Factor Models including ODD and CD symptoms

Models	χ^2^(df)	CFI	TLI	RMSEA (90% CI)	SRMR	AIC ^c^	BIC ^c^
Unidimensional CFA model	743.338* (119)	0.889	0.873	0.089 (0.083, 0.096)	0.087	21754.108	21983.058
Two-factor models ^a^ (ODD, CD)							
CFA	701.173* (118)	0.896	0.881	0.087 (0.081, 0.093)	0.081	21673.659	21907.098
ESEM	404.077* (103)	0.946	0.929	0.067 (0.060, 0.074)	0.058	21353.454	21654.230
Three-Factor models ^b^ (IRR, HS, CD)							
CFA	469.412* (116)	0.937	0.926	0.068 (0.062, 0.075)	0.070	21433.851	21676.268
ESEM	228.041* (88)	0.975	0.962	0.049 (0.041, 0.057)	0.045	21228.621	21596.736

*Note. N* = 658 self-ratings. ODD = oppositional defiant disorder, CD = conduct disorder; IRR = irritable; HS = headstrong; χ^2^  = chi-square test of model fit; *df* = degrees of freedom; CFI = comparative fit index; TLI = Tucker-Lewis index; RMSEA = root mean square error of approximation; CI = confidence interval; SRMR = standardized root mean square residual; AIC = Akaike information criterion; BIC = Bayesian information criterion. ^a^ ODD operationalized based on unidimensional ODD model. ^b^ IRR and HS operationalized based on multidimensional ODD model of Rowe et al. [25]. ^c^ Calculated using maximum likelihood estimation with robust standard errors (MLR) for continuous indicators. *p <.01

CD is often comorbid with ODD [37] and can be defined as a repetitive and persistent pattern of behavior that violates the rights of others or major age-appropriate societal norms or rules [8]. Although there is a close relationship between ODD and CD with overlapping risk factors [38], they are considered as two distinct disorders within the classification systems [8, 39], which is supported by empirical research [40]. Depending on the classification system, ODD can either be diagnosed together with CD (DSM-5) or considered part of CD when both sets of criteria are met (ICD-10).

While research has shown that ODD and CD dimensions can be distinguished from each other by factor analysis [11], it is questionable whether the typically used confirmatory factor analysis (CFA) adequately takes the high degree of interrelationship between the two disorders into account. For instance, CFA models are criticized because indicator variables are only supposed to load on their predefined latent factor, and cross-loadings between indicator variables and other latent factors are not allowed. In exploratory factor analysis (EFA), by contrast, cross-loadings are allowed, but are restricted with respect to the comparisons between different models [41, 42]. An innovative approach that combines the advantages of EFA (e.g., allowing cross-loadings) and CFA (e.g., a priori model specification; comparisons of different models by using model fit indices) is exploratory structural equation modeling (ESEM). Recent research has shown that ESEM can provide valuable information in order to disentangle symptoms of externalizing psychopathology and is a promising method to validate instruments which assess externalizing symptoms [43–45].

The aim of the present study was to gather new knowledge about the psychometric properties of self-rated ODD and CD symptoms, in particular regarding factorial validity. As recent research has highlighted the importance of validation studies in clinical samples, we decided to perform analyses in a clinical sample [46]. Using symptom ratings from adolescents aged between 11;0 and 17;11 years, we analyzed the factor structure of self-rated ODD and CD symptoms in two steps. First, we compared six different ODD models which were derived from previous research. Second, we examined the factor structure of the sub-dimensions of the preferred ODD model from the first step and an additional CD symptom dimension, resulting in final preferred models that include ODD and CD symptoms. Subsequently, we tested the measurement invariance of the final preferred models across age groups (≤ 13;11 years vs. ≥ 14;0 years) and informants (self-ratings vs. parent ratings; self-ratings vs. teacher ratings). Finally, we examined the internal consistencies as well as the convergent, divergent and discriminant validity indices of the self-rated symptom scales.

## Methods

### Measures

The *Symptom Checklist for Disruptive Behavior Disorders - Self-Rating* (SBB-SSV) was used to assess self-rated symptoms of ODD and CD in adolescents. The SBB-SSV is part of both the *Diagnostic System of Mental Disorders in Children and Adolescents based on the ICD-10 and DSM-IV* (DISYPS-II) [47] and the latest version of the *Diagnostic System of Mental Disorders in Children and Adolescents based on the ICD-10 and DSM-5* (DISYPS-III) [48]. The two versions collect identical ODD and CD items with one exception: the DISYPS-III combines two ODD items (*I am quick to anger*; *I have more frequent or stronger anger outbursts than others my age*) into one item (*I am quick to anger or have more frequent or stronger anger outbursts than others my age*). Therefore, the two items from the DISYPS-II were averaged for our analysis, thereby following the DSM-5-based nomenclature. As a result, we used eight items capturing ODD symptoms and 16 items capturing CD symptoms (see Table S1). In both versions, symptoms of ODD and CD were rated on a 4-point Likert scale ranging from 0 (*not at all*) to 3 (*very much*). In line with the diagnostic manuals, symptom ratings with more than 10% missing items were excluded from the data analyses [47, 48].

The *Symptom Checklist for Disruptive Behavior Disorders - Proxy rating* (FBB-SSV) was used to capture parent and teacher ratings of ODD and CD symptoms. The FBB-SSV is likewise part of the DISYPS-II [47] and DISYPS-III [48]. As with the SBB-SSV, the DISYPS-III combines the same two ODD items from the DISYPS-II into one item. Therefore, the two items of the older version were likewise averaged in parent and teacher ratings. With respect to the teacher ratings, three CD items did not have to be rated (B01A *physical fights with siblings*, B03 *cruel to animals*, B13 *stays out at night*), as teachers cannot typically evaluate these behaviors reliably. As a result, there were eight items capturing ODD symptoms from parents and teachers, 16 items capturing CD symptoms from parents, and 13 items capturing CD symptoms from teachers. In both versions, symptoms of ODD and CD were rated on a 4-point Likert scale ranging from 0 (*not at all*) to 3 (*very much*). The symptom checklists have shown mostly adequate psychometric properties in terms of validity and reliability in clinical (α = 0.67 − 0.93) and community samples (α = 0.71 − 0.90) [10, 12]. In line with the diagnostic manuals, symptom ratings with more than 10% missing items were excluded from the data analyses [47, 48].

The YSR [49] was used to assess self-rated emotional and behavioral problems of adolescents. The YSR consists of 112 items that can be aggregated into empirically based syndrome scales, broadband scales, and DSM-oriented scales. For our analysis, we used the eight syndrome scales (*Withdrawn*,* Somatic Complaints*,* Anxious/depressed*,* Social Problems*,* Thought Problems*,* Attention Problems*,* Rule-Breaking Behavior*,* Aggressive Behavior*) and two broadband scales (*Internalizing Problems*,* Externalizing Problems*). Items were rated on a 3-point scale (0 = *not true*, 1 = *somewhat/sometimes true*, 2 = *very/often true*). The eight syndrome and two broadband scales have shown mostly adequate psychometric properties in terms of validity and reliability in clinical (α = 0.65 − 0.89) and community samples (α = 0.68 − 0.89) [49]. In accordance with the test manual, ratings with more than eight missing items, with some exceptions (e.g., item on socially desirable behavior), were excluded. For included ratings, missing items were replaced with 0 [49].

### Sample and Procedure

The present sample consisted of adolescents referred to the outpatient unit of the Department of Child and Adolescent Psychiatry, Psychosomatics and Psychotherapy of the University Hospital of Cologne, Germany. We limited our sample to the data of adolescents aged 11;0 to 17;11 years at admission to treatment, who had less than 10% missing items on the SBB-SSV. In line with previous studies analyzing externalizing symptoms in clinically referred samples [10, 12], only data from participants with a primary diagnosis of ODD/CD and/or attention-deficit hyperactivity disorder (ADHD) were included. The diagnoses were assessed based on a semi-structured clinical interview for parents and adolescents, with diagnostic checklists from either the DISYPS-II [47] or DISYPS-III [48]. Since it is not possible to be diagnosed with both ODD and CD according to the ICD-10, for children who fulfilled the criteria for CD the diagnosis of ODD was excluded.

Our final sample consisted of 658 adolescents (age range 11;0–17;11 years, *M* = 12.59, *SD* = 1.43, 85% boys). Within the sample, *n* = 207 had a primary diagnosis of ODD/CD (ICD-10 codes F91 *Conduct disorders* or F92 *Mixed disorders of conduct and emotions*), *n* = 247 had a primary diagnosis of ODD/CD and ADHD (ICD-10 code F90.1 *Hyperkinetic conduct disorder*), and *n* = 204 had a primary diagnosis of ADHD (ICD-10 code F90 *Hyperkinetic disorder* except F90.1). YSR ratings were available from *n* = 642 adolescents. We further obtained symptom ratings from parents in *n* = 623 cases and from teachers in *n* = 511 cases.

### Data Analysis Plan

The examination of factor models and measurement invariance was conducted using Mplus 8.3 [50]. For descriptive analyses and calculations of internal consistency and convergent, divergent and discriminant validity, IBM SPSS Statistics for Windows version 27 was used [51].

#### Factor Structure of Self-Rated ODD Symptoms

First, we compared six correlated first-order ODD factor models using CFA in the sample of adolescents (see Fig. 1). The Stringaris and Goodman model [26] was not tested, as we assumed a minimum number of two indicator variables per factor [52]. Due to the ordinal structure of our data, we used the weighted least squares mean and variance adjusted estimator (WLSMV, delta parameterization), which is suggested for modeling ordinal data [53]. We had a small number of missing values for all ODD items (< 1% for each item, covariance coverage for each item was ≥ 0.992), and used pairwise missing deletion, the default option in Mplus for WLSMV. For individuals who completed the older version of the symptom checklist [47], averaging two items resulted in item values with decimal numbers for *n* = 191 of cases. As decimal numbers cannot be included in calculations of CFA in Mplus by using the WLSMV estimator, we decided to round up the item values for this single item (A01 *loses temper*). However, all models were also tested with rounded-off values and no notable differences emerged (see Table S2). To evaluate the global model fit of the six models, we used the chi-square test of model fit (χ^2^ test), the comparative fit index (CFI), the Tucker-Lewis index (TLI), the root mean square error of approximation (RMSEA) with 90% confidence intervals, and the standardized root mean square residual (SRMR). An excellent model fit was assumed if CFI and TLI > 0.95 and RMSEA and SRMR ≤ 0.05. For marginally acceptable model fit, CFI and TLI should be ≥ 0.90 and RMSEA and SRMR ≤ 0.08. The χ^2^ test should be non-significant (*p* >.01) [42]. In this study, we primarily relied on model fit indices of CFI, TLI, and SRMR, as χ^2^ tests are sensitive to sample size [54] and SRMR outperforms RMSEA when dealing with categorical data [55]. Furthermore, we calculated the Akaike information criterion (AIC) and the Bayesian information criterion (BIC) for each of the six models, which can be used to select the best fitting model among competing models [52]. As BIC and AIC cannot reported for WLSMV, we estimated the BIC and AIC by calculating the models using maximum likelihood estimation with robust standard errors (MLR) for continuous indicators and Monte Carlo simulation (1,500 integration points). A similar procedure has been used in other studies [44]. In general, the smaller the values of AIC and BIC, the more likely the model should be preferred [52]. As a rule of thumb, a difference of 10 in the BIC may indicate that the model with the lower value has the better model fit [56]. Furthermore, we examined the measurement quality of well-fitting ODD models by analyzing standardized factor loadings and correlations. Regarding factor loadings, we assumed minimum loadings of λ > 0.35 [42]. With respect to factor correlations, we assumed correlations of *r* >.80 to have poor discriminant validity [53].

#### Factor Structure of Self-Rated ODD and CD Symptoms

Second, we examined five correlated first-order CFA and ESEM models using ODD and CD symptoms. We calculated a CFA model (WLSMV, delta parameterization) and an ESEM model (WLSMV, theta parameterization, oblique target rotation) of the sub-dimensions from the preferred ODD model and included an additional CD symptom dimension. In addition, we calculated three further models: first, a unidimensional CFA model, where all ODD and CD items load onto one factor; second, a two-factor CFA model with a single ODD factor including all ODD items and a CD factor; and third, a two-factor ESEM model with a single ODD factor including all ODD items and a CD factor. In accordance with previous research [10, 12], we removed seven extremely skewed CD items with more than 95% percent of assessments indicated as 0 (*not present*). Finally, the CD factor consisted of nine items (B01A, B01B, B02, B04, B05, B06, B11, B13, B15; see Table S1). There was a small number of missing values for ODD and CD items (< 1% for each item, covariance coverage for each item was ≥ 0.992). We analyzed the model fit of CFA and ESEM models by examining χ2 tests, CFI, TLI, RMSEA, SRMR, AIC, and BIC. In general, ESEM models should be preferred over CFA models if they show better model fit indices, demonstrate reduced factor correlations, have strong loadings of indicator variables on their target factor, and display small to medium cross-loadings of indicator variables on non-target factors [57].

#### Measurement Invariance Across Age Groups and Informants

Third, the final preferred models from the second step were tested for measurement invariance (WLSMV estimator, theta parameterization) across age groups (≤ 13;11 years vs. ≥ 14;0 years) and informants (self-ratings vs. parent ratings; self-ratings vs. teacher ratings). In terms of the ESEM model, we used an invariance syntax generator for Mplus [58]. The basic idea of testing measurement invariance is a stepwise trimming strategy. First, the configural invariance model with a minimum of constraints was tested (same item-factor organization across groups). Subsequently, further constraints were imposed and metric invariance was tested (same factor loadings across groups). In a final step, even more constraints were imposed and scalar invariance was tested (same item thresholds across groups) [52]. To test for configural invariance, the same above-mentioned cut-off values of χ2 test, CFI, TLI, RMSEA, and SRMR were applied. To test for metric invariance and scalar invariance, we relied on changes in model fit indices. In terms of CFI, a difference of ≤ −0.01 was assumed to be evidence of measurement invariance [59]. Additionally, we assumed measurement invariance when observing a better fit of TLI and RMSEA [60], a change in SRMR of < 0.03 for metric invariance, and a change in SRMR of < 0.01 for scalar invariance [61].

#### Internal Consistency

Fourth, we evaluated the internal consistencies of self-rated ODD and CD scales by estimating Cronbach’s α [62] and McDonald`s ω [63] in the sample of adolescents. Values ≥ 0.70 were considered as acceptable [53]. Additionally, we examined item-total correlations and considered values of > 0.30 as acceptable [64]. Calculations were made for the sub-dimensions of the preferred ODD model from the first step, the ODD symptom scale including the full item pool (*ODD-Full Item Pool*, 8 items), the CD symptom scale excluding extremely skewed items with more than 95% percent of assessments indicated as 0 (*CD-Short Version*, 9 items), and the CD symptom scale including the full item pool (*CD-Full Item Pool*, 16 items).

#### Convergent and Divergent Validity

Fifth, we examined the convergent and divergent validity of the above-mentioned ODD and CD symptom scales by calculating Pearson product-moment correlations between the symptom scales with the *Internalizing Problems* scale, *Externalizing Problems* scale, and the eight syndrome scales of the YSR. Moreover, we tested for significant differences between pairs of correlations regarding ODD/CD scales and the *Internalizing Problems* scale versus ODD/CD scales and the *Externalizing Problems* scale based on z-transformation^1^[65].

#### Discriminant Validity

Sixth, we investigated the discriminant validity by comparing the levels of the above-mentioned ODD and CD symptom scales between the three diagnostic groups specified in the sample description: children with a diagnosis of ODD/CD (*n* = 207), children with a combined diagnosis of ODD/CD and ADHD (*n* = 247), and children with a diagnosis of ADHD (*n* = 204). This approach is in line with previous research evaluating ODD and CD symptoms in parent and teacher ratings [10, 12]. We conducted several analyses of variance (ANOVAs) with diagnostic group as between-subject variable and the different ODD and CD symptom scales as dependent variables. In case of violations of variance homogeneity (Levene’s test *p* <.05 and variance ratio > 1.5 [66]), we applied the robust Welch-ANOVA and the Games-Howell post-hoc test; otherwise, we used the classic one-way ANOVA and Tukey’s HSD post-hoc test [64].

## Results

### Factor Structure of Self-Rated ODD Symptoms

A total of six correlated first-order CFA models of ODD symptoms were calculated in the sample of adolescents (see Table 1). For CFI, TLI, and SRMR, the models postulated by Rowe et al. [25] and Aebi et al. [27] (CFI = 0.971–0.981, TLI = 0.957 − 0.968, SRMR = 0.035 − 0.041) showed excellent model fit indices. The model of Burke & Loeber [24] showed only marginally acceptable model fit indices (CFI = 0.948, TLI = 0.902, SRMR = 0.054). For SRMR, other models also showed at least an adequate fit (SRMR ≤ 0.08), whereas for CFI and TLI, none of the other models showed a marginally acceptable model fit in both indices together (CFI < 0.90 and TLI < 0.90). The χ^2^ tests were significant in all of the models (*p* <.01) and the RMSEA was not acceptable in any of the models (RMSEA > 0.08). The lowest AIC and BIC values were observed in the model of Burke & Loeber [24]. However, it should be kept in mind that AIC and BIC take a model’s complexity-parsimony into account, as reflected by degrees of freedom [54], and that the model of Burke & Loeber [24] only included six of the eight ODD items. As outlined above, the model showed only a marginally acceptable fit with respect to model fit indices, and other models revealed excellent model fit indices. For the models including all of the eight ODD items, the lowest AIC and BIC values were observed for the models of Aebi et al. [27] and Rowe et al. [25]. A difference > 10 in the BIC indicated that the model of Aebi et al. [27] yielded a better fit than the model of Rowe et al. [25]. Due to the excellent model fit in TLI, CFI, and SRMR of the models of Aebi et al. [27] and Rowe et al. [25], the superiority in AIC and BIC compared to all models including eight ODD items, and the content overlap of the two models (e.g., formulation of the same Irritable dimension), we selected both models for a closer inspection of factor loadings and correlations (see Tables S3-S4).

With respect to factor loadings, the models of Aebi et al. [27] and Rowe et al. [25] showed strong factor loadings of the indicator variables on their corresponding factors (λ *=* 0.58 − 0.88). In terms of factor correlations, the factors demonstrated strong intercorrelations in both models (*r* =.66 −.83). In particular, the strong correlation between the ODD-Hurtful factor and the ODD-Headstrong factor (*r* =.83) from the model of Aebi et al. [27] indicated that the two factors could be combined into one factor. Since the model of Rowe et al. [25] combines precisely these two factors into one factor, we decided to take the Rowe et al. [25] model into the next analysis step.

### Factor Structure of Self-Rated ODD and CD Symptoms

As a next step, we analyzed ODD and CD symptoms in the sample of adolescents by calculating five CFA and ESEM models (see Table 2). With respect to the three-factor CFA model (two sub-dimensions from model of Rowe et al. [25] plus one CD factor), we found marginally acceptable model fit indices (CFI = 0.937, TLI = 0.926, RMSEA = 0.068, SRMR = 0.070). However, the corresponding three-factor ESEM models revealed excellent model fit indices (CFI = 0.975, TLI = 0.962, RMSEA = 0.049, SRMR = 0.045). Unacceptable model fit indices (CFI < 0.90, TLI < 0.90, RMSEA > 0.08, SRMR > 0.08) were obtained for the unidimensional CFA model and the two-factor CFA model (single ODD dimension plus one CD factor). The corresponding two-factor ESEM model showed marginally acceptable model fit indices (CFI = 0.946, TLI = 0.929, RMSEA = 0.067, SRMR = 0.058). The χ^2^ tests were significant in all of the tested models (*p* <.01). In general, lower AIC and BIC values indicated better model fits of the ESEM models than their corresponding CFA models. Furthermore, the three-factor CFA and ESEM models outperformed their corresponding two-factor CFA and ESEM models with respect to AIC and BIC; therefore, we selected the three-factor models for a closer inspection of factor loadings and factor correlations (see Tables S5-S6).

A closer look at the factor loadings of the three-factor ESEM model revealed strong target factor loadings of the ODD-Irritable factor (λ = 0.72 − 0.83), no notable cross-loadings of the Irritable items on non-target factors (λ = −0.04 − 0.14), and only two items (A08 *spiteful / vindictive*; B01B *physical fights with other children*) demonstrating notable cross-loadings on the ODD-Irritable factor (λ = 0.40 − 0.43). However, with respect to the ODD-Headstrong factor, we found different indicator variables with low factor loadings on their corresponding target factors (λ ≤ 0.35). In particular, the two indicator variables (A06 *deliberately annoys others*, A08 *spiteful / vindictive*) showed lower factor loadings on their target factor (λ = − 0.02 − 0.20) than cross-loadings on their non-target CD factor (λ = 0.46 − 0.48) or ODD-Irritable factor (λ = 0.19 − 0.40). Furthermore, some items (e.g., A07 *blames others*, B04 *lies*, B11 *vandalism*) loaded moderately on their own target factor (λ = 0.31 − 0.52) and additionally loaded moderately on non-target factors (λ = 0.31 − 0.49). Despite these constraints of the three-factor ESEM model, most of the indicator variables showed at least adequate factor loadings to their target factors (λ > 0.35). A closer look at the factor loadings of the three-factor CFA model revealed well-defined factors with strong factor loadings (λ = 0.41 − 0.87).

With respect to the factor correlations, we found lower factor correlations in the three-factor ESEM model (*r* =.42 −.50) than in the three-factor CFA model (*r* =.69 −.91). In particular, the correlations between the ODD-Headstrong factor and CD factor in the three-factor CFA model revealed that the two factors could not be distinguished from each other (*r* =.91). Based on the aforementioned strengths and limitations, it was difficult to choose between the two three-factor models, so we decided to include both models in the next steps of analysis and to discuss the corresponding strengths and limitations later on.

### Measurement Invariance Across Age Groups and Informants

In terms of measurement invariance of the three-factor CFA and ESEM models across age groups (see Table S7), the configural models of both models showed measurement invariance through acceptable to excellent model fit indices (CFI = 0.940 − 0.974, TLI = 0.930 − 0.960, RMSEA = 0.049 − 0.065, SRMR = 0.052 − 0.077). The χ2 tests were significant in both models (*p* <.01). Furthermore, difference tests of model fit indices demonstrated measurement invariance for both models on a metric level (ΔCFI ≤ −0.01, equal or better fit of TLI and RMSEA, ΔSRMR < 0.03) and a scalar level (ΔCFI ≤ −0.01, equal or better fit of TLI and RMSEA, ΔSRMR < 0.01).

In terms of measurement invariance of the three-factor CFA and ESEM models across self-ratings and parent ratings (see Table S8), the configural models of both models showed measurement invariance through acceptable to excellent model fit indices (CFI = 0.931 − 0.955, TLI = 0.919 − 0.930, RMSEA = 0.075– 0.080, SRMR = 0.053 − 0.080). The χ2 tests were significant in both models (*p* <.01). Furthermore, difference tests of model fit indices demonstrated measurement invariance for both models on a metric level (ΔCFI ≤ −0.01, equal or better fit of TLI and RMSEA, ΔSRMR < 0.03), but not on a scalar level (ΔCFI > −0.01, worse fit of TLI and RMSEA).

In terms of measurement invariance of the three-factor CFA and ESEM models across self-ratings and teacher ratings (see Table S9), the configural models for both models showed measurement invariance through mostly acceptable to excellent model fit indices (CFI = 0.958 − 0.970, TLI = 0.947 − 0.948, SRMR = 0.048 − 0.068). The χ2 tests were significant in both models (*p* <.01). Furthermore, difference tests of model fit indices demonstrated measurement invariance for both models on a metric level (ΔCFI ≤ −0.01, equal or better fit of TLI and RMSEA, ΔSRMR < 0.03), but not on a scalar level (worse fit of TLI and RMSEA).

### Internal Consistency

The internal consistencies of self-rated ODD and CD symptom scales in the sample of adolescents were good for the *ODD-Full Item Pool* scale (α = 0.84; ω *=* 0.84) and the *ODD-Irritable* scale (α = 0.84; ω *=* 0.84; see Table S10). The *ODD-Headstrong* scale (α = 0.74; ω *=* 0.74) and the *CD-Full Item Pool* scale (α = 0.74, ω *=* 0.71) showed acceptable internal consistencies. However, four items of the latter scale (B01A *physical fights with siblings*, B03 *cruel to animals*, B10 *fire setting*, B12 *breaking in*) showed item-total correlations below the recommended cut-off (*r*_it_ ≤ 0.30). The *CD-Short Version* scale (α = 0.68; ω *=* 0.68) was marginally below the recommended cut-off (α *≥* 0.70).

### Convergent and Divergent Validity

In general, the ODD and CD symptom scales demonstrated weak to strong correlations with YSR externalizing scales (*Externalizing Problems*, *r* =.46 −.65; *Aggressive Behavior*, *r* =.47 −.63; *Rule-Breaking Behavior*, *r* =.27 −.48) and weak to moderate correlations with YSR internalizing scales (*Internalizing Problems*, *r* =.36 −.41; *Withdrawn*, *r* =.17 −.26; *Somatic Complaints*, *r* =.13 −.22; *Anxious/depressed*, *r* =.26 −.39; see Table S11). A comparison of correlation coefficients revealed that all ODD and CD symptom scales were significantly more strongly associated with the *Externalizing Problems* scale than with the *Internalizing Problems* scale (z = 3.09 – 9.70, all *p* <.01; see Table S12).

### Discriminant Validity

In terms of discriminant validity, the ANOVAs demonstrated significant differences between the three diagnostic groups (ODD/CD group, ODD/CD + ADHD group, ADHD group) across all ODD and CD symptom scales (*F* = 16.25 – 25.02, all *p* <.001; see Tables S13-S14). The post-hoc tests (see Table S15) demonstrated significantly higher levels on all symptom scales in the ODD/CD group compared to the ADHD group (mean difference = 0.05 – 0.42, all *p* <.05). Similarly, the ODD/CD + ADHD group showed significantly higher levels on all symptom scales compared to the ADHD only group (mean difference = 0.12 – 0.45, all *p* <.001). Furthermore, the post-hoc tests between the ODD/CD group and the ODD/CD + ADHD group revealed lower levels of reported symptoms in the ODD/CD group for the *ODD-Headstrong* scale, the *CD-Full Item Pool* scale, and the *CD-Short Version* scale (mean difference = -0.07 – -0.15, all *p* <.05).

## Discussion

The aim of this study was to gather new knowledge about the psychometric properties of self-rated ODD and CD symptoms. For this purpose, we examined symptom ratings of a large clinical sample of 658 adolescents aged 11;0–17;11 years (85% boys). First, we compared six ODD models by using CFA. The results indicated that a separation into different ODD sub-dimensions seems to be reasonable, as the unidimensional DSM-5 model only showed partially adequate model fit indices, and different multidimensional models outperformed the unidimensional model with respect to model fit indices. These results are in line with empirical research demonstrating that ODD symptomatology is multidimensional rather than unidimensional [32, 35, 36]. However, there is disagreement about which multidimensional model is superior to other models across different samples (e.g., clinical, community) and informants (e.g., teachers, parents, adolescents). Our study aimed to shed light on the multidimensionality of ODD symptomatology by focusing on self-ratings from a clinical sample of adolescents. When comparing multidimensional ODD models against each other, the three-factor model of Aebi et al. [27] and the two-factor model Rowe et al. [25] were identified as two well-fitting models with respect to model fit indices and factor loadings. Therefore, in contrast to a previous study that included self-ratings from a community sample [36], our results indicated that not only the model of Aebi et al. [27], but also the model of Rowe et al. [25], might be a well-defined ODD model in a clinical sample. Due to the strong correlations between the ODD-Hurtful factor and the ODD-Headstrong factor (*r* >.80) in Aebi et al.’s model [27], we decided to prefer the model of Rowe et al. [25], which combines these two factors into one.

Second, we examined the factor structure of self-rated ODD and CD symptoms using CFA and ESEM. We preferred the three-factor CFA and ESEM models (two sub-dimensions from the model of Rowe et al. [25] plus one CD factor), as these models outperformed their corresponding two-factor models and the unidimensional model with respect to model fit indices. When comparing the two three-factor models, the three-factor ESEM model outperformed the corresponding CFA model with respect to model fit indices. Furthermore, we observed lower factor correlations in the three-factor ESEM model than in the three-factor CFA model. These findings are consistent with previous research analyzing ESEM models in parent and teacher ratings of externalizing psychopathology [44, 45] and indicate that ESEM models can provide a valuable approach to address the high degree of interrelationship between ODD and CD symptomatology. Regarding the three-factor ESEM model, the ODD-Irritable factor emerged as well-defined (strong factor loadings, few substantial cross-loadings), underlining that based on self-ratings, the ODD-Irritable dimension is well separable not only from other ODD sub-dimensions but also from a CD dimension. However, there were limitations in differentiating the ODD-Headstrong dimension from the CD dimension, as indicated by low loadings on their own target factor and high cross-loadings on non-target factors for some indicator variables. In particular, the items A06 *deliberately annoys others* and A08 *spiteful / vindictive* were strongly related to the CD dimension, which limited the validity of the three-factor ESEM model. These results may indicate on the one hand that these two items should be considered as a separate factor, in accordance with Aebi et al.’s [27] model, which might have a unique association with CD symptomatology, but on the other hand may be explained by the skewed distribution of the two ODD items, which is similar to those of the CD items. Moreover, some items loaded moderately on their own target factor and additionally loaded moderately on non-target factors (e.g., A07 *blames others*, B04 *lies*, B11 *vandalism*), potentially indicating that these items represent *linking symptoms* between these factors. A more detailed examination of these results could be performed using network analyses, which focus on links between dimensions on a symptom level [67]. With regard to the three-factor CFA models, we found well-defined factor loadings. However, strong correlations between the ODD-Headstrong dimension and CD dimension indicated difficulties in distinguishing the factors from each other. These correlations may indicate that more complex factor CFA/ESEM models are required to disentangle ODD and CD symptoms, which will be further discussed below. All in all, there is no easy answer as to whether the three-factor ESEM or CFA model should be preferred, as both models showed advantages and limitations. The integration of cross-loadings to account for the interrelationships of ODD and CD symptomatology, mostly excellent model fit indices, lower AIC and BIC values, and lower factor correlations spoke in favor of the ESEM model. However, greater parsimony and well-defined factor loadings spoke in favor of the CFA model. Therefore, we decided to test measurement invariance for both models.

Third, we examined measurement invariance of the three-factor CFA and ESEM models across age groups (≤ 13;11 years vs. ≥ 14;0 years) and informants (self-ratings vs. parent ratings; self-ratings vs. teacher ratings). Regarding age groups, we found configural, metric, and scalar measurement invariance for the three-factor CFA and ESEM models, indicating that the two age groups showed similarities regarding item-factor organization, factor loadings, and item thresholds. These results are in line with previous studies investigating measurement invariance of ODD and CD dimensions across age groups and over time, based on ratings by different informants in community samples [3, 21, 31, 35]. However, there are also some findings to the contrary. For instance, using items of the YSR, Evans et al. [46] showed that metric and scalar measurement invariance could not be achieved across age groups in a clinical sample. With respect to measurement invariance analyses across informants, our results demonstrated that configural and metric invariance could be assumed for the three-factor ESEM and CFA models across self-ratings and parent ratings as well as across self-ratings and teacher ratings. Thus, it might be concluded that the item-factor organization and factor loadings were equal, whereas the individual symptom severity differed across informants. Our study is in line with previous research indicating a lack of scalar invariance across informants with respect to externalizing psychopathology [44, 68], and provides further evidence that ratings of children’s behavior vary meaningfully across informants (adolescents, parents, teachers). It is important to mention that current research strongly questions whether measurement invariance should be conducted across informants, mainly because a large body of evidence has shown that informant discrepancies contain domain-relevant information, and measurement invariance techniques do not properly take this fact into account [69]. At this time, it is unclear how this debate about informant discrepancies and the application of measurement invariance will evolve. Based on our results, we do not question that discrepancies between informants do have informational value, and the objective of our study was accordingly not to exclude non-invariant ODD or CD symptoms.

Fourth and fifth, we examined internal consistencies as well as the convergent and divergent validity indices of ODD and CD scales. Although the ODD-Irritable dimension was measured with only three items, which is typical when assessing irritability in the context of ODD symptom items [22], we found good internal consistencies and adequate inter-item correlations. In addition, higher correlations with YSR externalizing scales than with YSR internalizing scales revealed convergent and divergent validity of the ODD-Irritable dimension. These results suggest - supported by the good differentiation of the ODD-Irritable factor from other ODD and CD factors in factor analyses - that symptom ratings of adolescents can provide a reliable and valid assessment method for an ODD-Irritable dimension. With respect to the ODD-Headstrong dimension, our analyses demonstrated acceptable internal consistencies as well as mostly adequate convergent and divergent validity indices. All in all, these results imply that the use of an *ODD-Irritable* scale and an *ODD-Headstrong* scale (measured via five items) in self-ratings from a clinical sample can provide an accurate and valid assessment of ODD symptomatology. These results are consistent with the strong support found for an ODD-Irritable dimension, moderate support for an ODD-Headstrong/Defiant dimension, and weak support for an ODD-Hurtful dimension [22]. Regarding internal consistencies of the CD symptoms, the *CD-Short Version* scale was below the recommended cut-off and the *CD-Full Item Pool* scale showed low item-total correlations with respect to four items. Although convergent and divergent validity indices appeared to be mostly adequate, our results indicated only limited psychometric properties of the CD dimension in self-ratings from a clinical sample. A recent study by Thöne et al. [13], evaluating a semi-structured parent interview of externalizing disorders in a large clinical sample, reached similar findings. In this study, the low internal consistency was explained by the heterogeneity of the CD items and the skewed distribution of the items.

Sixth, we examined the discriminant validity indices of ODD and CD symptom scales. Consistent with previous research based on parent and teacher ratings [10, 12], higher symptom levels in the two groups of children with ODD/CD compared to the ADHD group without an additional ODD/CD diagnosis indicated a good ability of the self-rated symptom scales in differentiating between children with ODD/CD and those with ADHD only. These findings support the idea that self-ratings can provide valuable diagnostic information [5, 17–19]. However, in contrast to previous research [10, 12], ratings differed significantly between the ODD/CD group and the ODD/CD + ADHD group, with the latter group reporting higher symptom levels on three scales. This discrepancy might be due to differences in sample characteristics, as we examined ratings of adolescents and did not collect data from children aged 4–10 years. Another possible explanation might be that adolescents with ODD/CD (but without ADHD) underreport their ODD/CD symptoms.

Several limitations of the present study should be mentioned. First, the difficulties that emerged in differentiating the ODD and CD dimensions from each other via first-order CFA and ESEM models might indicate that more complex factor CFA/ESEM models (e.g., higher-order models, bifactor models, bifactor s-1 models) could have provided more accurate results. A large number of previous studies have already demonstrated that such models contribute greatly to disentangling symptoms of externalizing psychopathology in children and adolescents across informants (e.g., parents, teacher, clinicians) and samples (e.g., clinical, community) [44, 70, 71]. Moreover, different studies focusing exclusively on the sub-dimensions of ODD have shown that higher-order and bifactor models could provide well-fitting models [29, 32, 33, 72]. For instance, Burke et al. [72] demonstrated that a bifactor model based on dimensions of Rowe et al.’s model [25] outperformed unidimensional and correlated/orthogonal first-order models in five large community samples. As recent studies have been limited to parent and teacher ratings [33, 72], an investigation of higher-order or bifactor CFA/ESEM models of ODD and CD symptoms in self-ratings might have provided further meaningful insights. However, we explicitly focused on first-order CFA/ESEM models as we wanted to focus on initial insights into the mutual correlations among ODD and CD dimensions and to provide a starting point for further factor-analytic investigations of bifactor or higher-order CFA/ESEM models in self-ratings.

Second, it was not possible to test for measurement invariance across gender using the WLSMV estimator for five items (A07 *blames others*, B02 *bullies*,* threatens*,* or intimidates*, B05 *steals without confrontation*, B06 *uses weapons in fights*, B11 *vandalism*), as not all response categories were completed in the subgroup of girls.

Third, as the main aim of our study was not to examine informant differences, we limited our analyses to testing measurement invariance across informants. At this point, we would like to encourage future research to include self-rated symptoms of ODD and CD when analyzing informant discrepancies. Promising statistical approaches may be, for example, latent class analyses [73] or indicator-specific trait-factor correlations [74].

Considering the implications for clinical practice, our study provides support for the use of self-rated ODD symptom scales. It is important to note that self-ratings should be implemented within a multi-informant approach [4, 5], and their validity may be limited due to underreporting [15]. However, one major advantage of self-ratings is that adolescents are the only informants who are truly informed about all events and social interactions in which ODD and CD symptoms may occur. Whether or to what extent self-rated symptoms can provide additional diagnostic value cannot be answered based on our study results. When self-ratings are used in clinical practice, ODD symptomatology can at least be divided into two different subdimensions: a more affective dimension (e.g., irritability) and a more behavioral dimension (e.g., defiant/headstrong). In particular, an ODD-Irritable dimension seemed to be well distinguishable from other ODD and CD dimensions and showed good internal consistency in self-ratings. Therefore, self-ratings may help clinicians to diagnose ODD with and without chronic irritability according to the *International Classification of Diseases 11th Revision* (ICD-11) [39]. If a more behavioral dimension is used in clinical practice, our results support the inclusion of all other five ODD items beyond the three irritable items. Furthermore, the use of CD scales is limited due to low internal consistencies and the very heterogeneous item pool.

### Summary

This study provided insights into the psychometric properties of self-rated ODD and CD symptoms from a large clinical sample of adolescents. A main focus was on examining the factor structure. In a first step, we demonstrated the factorial validity of the ODD model of Rowe et al. [25] using CFA. In a second step, using CFA and ESEM, we examined the differentiation between the sub-dimensions of the ODD model of Rowe [25] and an additional CD symptom dimension, resulting in two final preferred three-factor models. Measurement invariance analyses of both final preferred models across age groups (≤ 13;11 years vs. ≥ 14;0 years) and informants (self-ratings vs. parent ratings, self-ratings vs. teacher ratings) supported similarities of the factor structure in the different subgroups. Furthermore, internal consistencies were acceptable to good for self-rated ODD symptom scales, but showed limited support for CD symptom scales. The convergent and divergent validity of self-rated ODD/CD symptom scales were supported using scales of the YSR. The self-rated symptom scales demonstrated discriminant validity by differentiating between children with ODD/CD and those with ADHD only.

## Electronic Supplementary Material

Below is the link to the electronic supplementary material.

Supplementary Material 1

## Data Availability

Data and codes are available upon reasonable request.

## Footnotes

1 Detailed instructions can be found on the IBM SPSS STATISTICS support homepage (https://www.ibm.com/support/pages/testing-differences-between-dependent-correlations).
