# Supplementary material for: Self-Rated Symptoms of Oppositional Defiant Disorder and Conduct Disorder: Factor Structure, Reliability, and Validity in a Clinical Sample of Adolescents
Source: Child Psychiatry Hum Dev. 2024 Dec 11;56(4):1147–60. doi: 10.1007/s10578-024-01802-2 (PMC12289755; doi:10.1007/s10578-024-01802-2)
Supplement: Supplementary file 1 — Supplementary Material 1 [file 10578_2024_1802_MOESM1_ESM.docx]

**Online Supplement**

**Table S1**

*Item Descriptions and Descriptive Statistics of Self-Rated ODD and CD Items*

| No. | Item Description ^a^ | *n* | *M* | *SD* |
| --- | --- | --- | --- | --- |
| ODD symptoms | |  |  |  |
| A01 | Loses temper | 657 | 1.26 | 0.92 |
| A02 | Touchy / easily annoyed | 658 | 1.43 | 1.06 |
| A03 | Angry / resentful | 658 | 1.24 | 1.00 |
| A04 | Argues with adults | 654 | 1.05 | 0.89 |
| A05 | Defies requests | 657 | 1.24 | 0.85 |
| A06 | Deliberately annoys others | 656 | 0.71 | 0.82 |
| A07 | Blames others | 658 | 0.68 | 0.83 |
| A08 | Spiteful / vindictive | 657 | 0.58 | 0.87 |
| CD symptoms | |  |  |  |
| B01A | Physical fights with siblings | 658 | 1.00 | 0.96 |
| B01B | Physical fights with other children | 658 | 0.64 | 0.77 |
| B02 | Bullies, threatens, or intimidates | 657 | 0.28 | 0.60 |
| B03 | Cruel to animals | 657 | 0.05 | 0.30 |
| B04 | Lies | 658 | 0.68 | 0.85 |
| B05 | Steals without confrontation | 657 | 0.14 | 0.47 |
| B06 | Uses weapons in fights | 658 | 0.10 | 0.43 |
| B07 | Cruel to people | 658 | 0.05 | 0.29 |
| B08 | Steals with confrontation | 658 | 0.04 | 0.30 |
| B09 | Sexual assault | 655 | 0.01 | 0.16 |
| B10 | Fire setting | 658 | 0.02 | 0.21 |
| B11 | Vandalism | 658 | 0.09 | 0.38 |
| B12 | Breaking in | 658 | 0.02 | 0.17 |
| B13 | Stays out at night | 657 | 0.20 | 0.54 |
| B14 | Runs away from home overnight | 658 | 0.05 | 0.31 |
| B15 | Truancy | 657 | 0.10 | 0.40 |

*Note.* *N* = 658; ODD = oppositional defiant disorder; CD = conduct disorder.

^a^ Abbreviations of the original items.

**Table S2**

*Goodness-of-Fit Indices and Information Criteria of the ODD Factor Models (Rounded off)*

| Models | χ^2^(*df*) | CFI | TLI | RMSEA (90% CI) | SRMR | AIC ^a^ | BIC ^a^ |
| --- | --- | --- | --- | --- | --- | --- | --- |
| Unidimensional model [8] | 338.389* (20) | .922 | .890 | .156 (.141, .170) | .062 | 12382.906 | 12490.647 |
| Two-factor models | | | | |  | | |
| Burke & Loeber [24] | 199.583* (8) | .945 | .898 | .191 (.168, .214) | .054 | 9409.740 | 9495.035 |
| Rowe et al. [25] | 150.996* (19) | .968 | .952 | .103 (.088, .118) | .043 | 12237.739 | 12349.969 |
| Jungersen & Lonigan [35] | 329.920* (19) | .923 | .887 | .158 (.143, .173) | .061 | 12380.100 | 12492.330 |
| Three-factor models |  |  |  |  |  |  |  |
| Aebi et al. [27] | 102.598* (17) | .979 | .965 | .087 (.072, .104) | .035 | 12210.663 | 12331.872 |
| Burke et al. [28] | 272.185* (17) | .937 | .897 | .151 (.136, .167) | .053 | 12315.893 | 12437.101 |
| *Note.* *N* = 658 self-ratings. χ^2^ = chi-square test of model fit; *df* = degrees of freedom; CFI = comparative fit index; TLI = Tucker-Lewis index; RMSEA = root mean square error of approximation; CI = confidence interval; SRMR = standardized root mean square residual; AIC = Akaike information criterion; BIC = Bayesian information criterion. ^a^ Calculated using maximum likelihood estimation with robust standard errors (MLR) for continuous indicators. **p* < .01. | | | | | | | |

**Table S3**

*Standardized Factor Loadings (Standard Error) of the ODD Factor Models from Rowe et al. [25] and Aebi et al. [27]*

| Symptoms | Rowe et al. | | Aebi et al. | | |
| --- | --- | --- | --- | --- | --- |
|  | Irritable | Headstrong | Irritable | Headstrong | Hurtful |
| A01 Loses temper | .83 (.02) |  | .83 (.02) |  |  |
| A02 Touchy / easily annoyed | .83 (.02) |  | .83 (.02) |  |  |
| A03 Angry / resentful | .88 (.02) |  | .88 (.02) |  |  |
| A04 Argues with adults |  | .68 (.03) |  | .71 (.03) |  |
| A05 Defies requests |  | .75 (.03) |  | .81 (.03) |  |
| A06 Deliberately annoys others |  | .68 (.03) |  |  | .69 (.04) |
| A07 Blames others |  | .58 (.04) |  | .62 (.04) |  |
| A08 Spiteful / vindictive |  | .70 (.03) |  |  | .72 (.03) |
| *Note.* All factor loadings are significant (*p* < .001). | | | | | |

**Table S4**

*Standardized Factor Correlations of the ODD Factor Models of Rowe et al. [25] and Aebi et al. [27]*

|  | Irritable | Headstrong |
| --- | --- | --- |
| Irritable | - | .74 |
| Headstrong | .66 | - |
| Hurtful | .80 | .83 |
| *Note.* Factor correlations of Rowe et al.’s model above the diagonal; factor correlations of Aebi et al.’s model below the diagonal. All factor correlations are significant (*p* < .001). | | |

**Table S5**

*Standardized Factor Loadings (Standard Error) of the Three-Factor CFA Model and the Three-Factor ESEM Model*

| Symptoms | Three-factor CFA model  ^a^ | | | Three-factor ESEM model  ^a,b^ | | |
| --- | --- | --- | --- | --- | --- | --- |
|  | IRR | HS | CD | IRR | HS | CD |
| A01 Loses temper | .84 (.02)*** |  |  | **.72 (.03)***** | .14 (.04)*** | .04 (.04) |
| A02 Touchy / easily annoyed | .82 (.02)*** |  |  | **.82 (.03)***** | .08 (.04)* | -.04 (.03) |
| A03 Angry / resentful | .87 (.02)*** |  |  | **.83 (.03)***** | .13 (.03)*** | -.03 (.03) |
| A04 Argues with adults |  | .63 (.03)*** |  | .13 (.05)** | **.66 (.06)***** | -.02 (.05) |
| A05 Defies requests |  | .72 (.02)*** |  | .12 (.04)** | **.77 (.06)***** | .02 (.04) |
| A06 Deliberately annoys others |  | .72 (.03)*** |  | .19 (.05)*** | **.20 (.06)**** | .48 (.06)*** |
| A07 Blames others |  | .62 (.03)*** |  | .04 (.05) | **.39 (.06)***** | .34 (.06)*** |
| A08 Spiteful / vindictive |  | .72 (.03)*** |  | .40 (.05)*** | **-.02 (.06)** | .46 (.05)*** |
| B01A Physical fights with siblings |  |  | .43 (.04)*** | .10 (.06) | .05 (.06) | **.35 (.07)***** |
| B01B Physical fights with other children |  |  | .76 (.03)*** | .43 (.05)*** | .03 (.05) | **.40 (.05)***** |
| B02 Bullies, threatens, or intimidates |  |  | .72 (.04)*** | .18 (.05)*** | -.09 (.06) | **.72 (.06)***** |
| B04 Lies |  |  | .59 (.03)*** | -.09 (.05) | .49 (.05)*** | **.31 (.06)***** |
| B05 Steals without confrontation |  |  | .59 (.05)*** | -.06 (.07) | .14 (.07) | **.59 (.09)***** |
| B06 Uses weapons in fights |  |  | .44 (.07)*** | -.10 (.08) | -.11 (.07) | **.69 (.09)***** |
| B11 Vandalism |  |  | .69 (.06)*** | -.02 (.09) | .31 (.09)** | **.52 (.08)***** |
| B13 Stays out at night |  |  | .41 (.06)*** | -.15 (.07)* | .06 (.08) | **.55 (.08)***** |
| B15 Truancy |  |  | .44 (.07)*** | -.20 (.08)* | -.04 (.09) | **.71 (.10)***** |
| *Note.* IRR = irritable; HS = headstrong; CD = conduct disorder. ^a^ IRR and HS operationalized based on multidimensional ODD model of Rowe et al. [25]. ^b^ Target factor loadings in ESEM model are printed in bold. **p* < .05; ***p* < .01; ****p* < .001. | | | | | | |

**Table S6**

*Standardized Factor Correlations of the Three-Factor CFA Model and the Three-Factor ESEM Model*

|  | Irritable | Headstrong | Conduct Disorder |
| --- | --- | --- | --- |
| Irritable | - | .74 | .69 |
| Headstrong | .42 | - | .91 |
| Conduct Disorder | .48 | .50 | - |
| *Note.* Factor correlations of the three-factor CFA model above the diagonal; factor correlations of the three-factor ESEM model below the diagonal. All factor correlations are significant (*p* < .001). | | | |

**Table S7**

*Measurement Invariance of the Three-Factor CFA/ESEM Models Across Age Groups*

| Models | χ^2^ *(df)* | CFI | TLI | RMSEA (90% CI) | SRMR |
| --- | --- | --- | --- | --- | --- |
| Three-Factor CFA model | | | | | |
| Configural | 554.110* (232) | .940 | .930 | .065 (.058, .072) | .077 |
| Metric | 513.881* (246) | .950 | .945 | .058 (.051, .065) | .081 |
| Scalar | 577.670* (294) | .947 | .951 | .054 (.048, .061) | .082 |
| Three-Factor ESEM model | | | | | |
| Configural | 316.800* (176) | .974 | .960 | .049 (.040, .058) | .052 |
| Metric | 350.025* (218) | .976 | .969 | .043 (.034, .051) | .057 |
| Scalar | 372.927* (249) | .977 | .975 | .039 (.030, .047) | .059 |
| *Note.* Age ≤ 13;11 years (*n* = 562), age ≥ 14;0 (*n* = 96). χ^2^ = chi-square test of model fit; *df* = degrees of freedom; CFI = comparative fit index; TLI = Tucker-Lewis index; RMSEA = root mean square error of approximation; CI = confidence interval; SRMR = standardized root mean square residual. **p* < .01. | | | | | |

**Table S8**

*Measurement Invariance of the Three-Factor CFA/ESEM Models Across Self-Ratings and Parent Ratings*

| Models | χ^2^ *(df)* | CFI | TLI | RMSEA (90% CI) | SRMR |
| --- | --- | --- | --- | --- | --- |
| Three-Factor CFA model | | | | | |
| Configural | 1192.621* (232) | .931 | .919 | .080 (.076, .085) | .080 |
| Metric | 1007.751* (246) | .945 | .939 | .070 (.065, .074) | .083 |
| Scalar | 1468.303* (294) | .915 | .922 | .079 (.075, .083) | .085 |
| Three-Factor ESEM model | | | | | |
| Configural | 803.306* (176) | .955 | .930 | .075 (.069, .080) | .053 |
| Metric | 706.767* (218) | .965 | .956 | .059 (.054, .064) | .057 |
| Scalar | 881.901* (249) | .954 | .950 | .063 (.059, .068) | .062 |
| *Note.* Self-ratings (*n* = 658), parent ratings (*n* = 623). χ^2^ = chi-square test of model fit; *df* = degrees of freedom; CFI = comparative fit index; TLI = Tucker-Lewis index; RMSEA = root mean square error of approximation; CI = confidence interval; SRMR = standardized root mean square residual. **p* < .01. | | | | | |

**Table S9**

*Measurement Invariance of the Three-Factor CFA/ESEM Models Across Self-Ratings and Teacher Ratings*

| Models | χ^2^ *(df)* | CFI | TLI | RMSEA (90% CI) | SRMR |
| --- | --- | --- | --- | --- | --- |
| Three-Factor CFA model | | | | | |
| Configural | 939.106* (148) | .958 | .948 | .096 (.090, .102) | .068 |
| Metric | 790.407* (159) | .966 | .961 | .082 (.077, .088) | .076 |
| Scalar | 1233.374* (198) | .945 | .949 | .095 (.090, .100) | .080 |
| Three-Factor ESEM model | | | | | |
| Configural | 670.967* (104) | .970 | .947 | .097 (.090, .104) | .048 |
| Metric | 542.981* (137) | .978 | .971 | .071 (.065, .078) | .054 |
| Scalar | 722.961* (162) | .970 | .966 | .077 (.071, .083) | .063 |
| *Note.* Self-ratings (*n* = 658); teacher ratings (*n* = 511). χ^2^ = chi-square test of model fit; *df* = degrees of freedom; CFI = comparative fit index; TLI = Tucker-Lewis index; RMSEA = root mean square error of approximation; CI = confidence interval; SRMR = standardized root mean square residual. Three items (B01A: Physical fights with siblings, B03: Cruel to animals, B13: Stays out at night) were omitted as they did not have to be assessed by teachers. One item (B05: Steals without confrontation) was omitted as it was extremely skewed (more than 95% percent of assessments indicated as 0) in teacher ratings.**p* < .01. | | | | | |

**Table S10**

*Internal Consistencies of Self-Rated ODD and CD Symptom Scales*

| Symptom Scale | *n* | No. of items | *M* | *SD* | α | ω | *r*_it min-max_ |
| --- | --- | --- | --- | --- | --- | --- | --- |
| ODD-Full Item Pool | 650 | 8 | 1.02 | 0.62 | .84 | .84 | .50 - .68 |
| ODD-Irritable | 657 | 3 | 1.31 | 0.87 | .84 | .84 | .69 - .72 |
| ODD-Headstrong | 651 | 5 | 0.85 | 0.60 | .74 | .74 | .50 - .60 |
| CD-Short Version | 654 | 9 | 0.36 | 0.33 | .68 | .68 | .31 - .48 |
| CD-Full Item Pool | 650 | 16 | 0.22 | 0.23 | .74 | .71 | .22 - .52 |
| *Note.* ODD = oppositional defiant disorder; CD = conduct disorder; α = Cronbach’s α; ω = McDonald’s ω; *r*_it_ = item-total correlations. | | | | | | | |

**Table S11**

*Correlations of ODD and CD Symptom Scales with YSR Scales*

|  | ODD | | | CD | |
| --- | --- | --- | --- | --- | --- |
|  | Full Item Pool | IRR | HS | Short Version | Full Item Pool |
| Syndrome scales | | | | | |
| Withdrawn | .26 | .24 | .22 | .18 | .17 |
| Somatic Complaints | .22 | .19 | .19 | .15 | .13 |
| Anxious/depressed | .39 | .37 | .32 | .29 | .26 |
| Social Problems | .29 | .28 | .24 | .22 | .20 |
| Thought Problems | .19 | .17 | .17 | .17 | .17 |
| Attention Problems | .36 | .28 | .35 | .30 | .26 |
| Rule-Breaking Behavior | .42 | .27 | .46 | .48 | .46 |
| Aggressive Behavior | .63 | .48 | .62 | .52 | .47 |
| Broadband scales |  |  |  |  |  |
| Internalizing Problems | .41 | .36 | .36 | .36 | .36 |
| Externalizing Problems | .63 | .46 | .65 | .59 | .55 |
| *Note.* *n* = 634 - 642 self-ratings. ODD = oppositional defiant disorder, CD = conduct disorder; IRR = irritable; HS = headstrong. All correlations are significant (*p* < .001). | | | | | |

**Table S12**

*Differences of Correlation Coefficients (self-rated ODD/CD Scales with Externalizing Problems vs. self-rated ODD/CD Scales with Internalizing Problems)*

| Symptom Scales | *r* | | diff *_r_* | *z* |
| --- | --- | --- | --- | --- |
|  | Externalizing  Problems | Internalizing  Problems |  |  |
| ODD-Full Item Pool | .63 | .41 | .22 | 7.42*** |
| ODD-Irritable | .46 | .36 | .10 | 3.09*** |
| ODD-Headstrong | .65 | .36 | .29 | 9.70*** |
| CD-Short Version | .59 | .36 | .23 | 7.47*** |
| CD-Full Item Pool | .55 | .36 | .19 | 5.97*** |
| *Note.*  *n* = 635 - 641. ODD = oppositional defiant disorder; CD = conduct disorder. **p* < .05; ***p* < .01; ****p* < .001. | | | | |

**Table S13**

*Means and Standard Deviations of Self-rated ODD/CD Scales for the Three Diagnostic Groups*

| Symptom Scales | ODD/CD | | |  | ODD/CD+ADHD | | |  | ADHD | | | | |
| --- | --- | --- | --- | --- | --- | --- | --- | --- | --- | --- | --- | --- | --- |
|  | *n* | *M* | *SD* |  | *n* | *M* | *SD* |  | *n* | | *M* | *SD* | |
| ODD-Full Item Pool | 206 | 1.07 | 0.60 |  | 242 | 1.18 | 0.65 |  | 202 | 0.78 | | | 0.53 |
| ODD-Irritable | 207 | 1.43 | 0.84 |  | 246 | 1.46 | 0.88 |  | 204 | 1.01 | | | 0.80 |
| ODD-Headstrong | 206 | 0.86 | 0.57 |  | 243 | 1.01 | 0.64 |  | 202 | 0.65 | | | 0.50 |
| CD-Short Version | 207 | 0.34 | 0.29 |  | 245 | 0.45 | 0.41 |  | 202 | 0.27 | | | 0.24 |
| CD-Full Item Pool | 206 | 0.21 | 0.20 |  | 245 | 0.28 | 0.29 |  | 199 | 0.16 | | | 0.14 |
| *Note.* ODD = oppositional defiant disorder; CD = conduct disorder; ADHD = attention-deficit hyperactivity disorder. | | | | | | | | | | | | | |

**Table S14**

*Variance Homogeneity Tests and ANOVAs for Self-rated ODD/CD Scales and Between-subject Variable Diagnostic Group*

| Symptom Scales |  | Levene Test | |  | Variance Ratio |  | Anova | |
| --- | --- | --- | --- | --- | --- | --- | --- | --- |
|  |  | *F (df_1,_ df_2_)* | *p* |  | *Var_max -_ Var_min_* |  | *F(df_1,_ df_2_)* | *p* |
| ODD-Full Item Pool |  | 4.37 (2, 647) | .013 |  | 1.47 |  | 25.02^a^ (2, 647) | <.001 |
| ODD-Irritable |  | 2.06 (2, 654) | .128 |  | 1.21 |  | 18.75^a^  (2, 654) | <.001 |
| ODD-Headstrong |  | 6.62 (2, 648) | .001 |  | 1.62 |  | 22.51^b^  (2, 431) | <.001 |
| CD-Short Version |  | 14.66 (2, 651) | <.001 |  | 2.84 |  | 16.25^b^  (2, 429) | <.001 |
| CD-Full Item Pool |  | 16.18 (2, 647) | <.001 |  | 4.05 |  | 16.60^b^  (2, 419) | <.001 |
| *Note.* ^a^ One-way ANOVA. ^b^ Welch-ANOVA. | | | | | | | | |

**Table S15**

*Post-hoc Tests for Self-rated ODD/CD Scales and the Three Diagnostic Groups*

| Symptom Scales | Group 1 | Group 2 | Mean Difference (95% CI) ^c^ | *p* |
| --- | --- | --- | --- | --- |
| ODD-Full Item Pool ^a^ | ODD/CD | ODD/CD+ADHD | -.10 (-.24, .03) | .167 |
|  | ODD/CD | ADHD | .29 (.15, .43) | <.001 |
|  | ODD/CD+ADHD | ADHD | .40 (.26, .53) | <.001 |
| ODD-Irritable ^a^ | ODD/CD | ODD/CD+ADHD | -.04 (-.22, .15) | .894 |
|  | ODD/CD | ADHD | .42 (.22, .61) | <.001 |
|  | ODD/CD+ADHD | ADHD | .45 (.26, .64) | <.001 |
| ODD-Headstrong ^b^ | ODD/CD | ODD/CD+ADHD | -.15 (-.28, -.01) | .026 |
|  | ODD/CD | ADHD | .21 (.09, .33) | <.001 |
|  | ODD/CD+ADHD | ADHD | .36 (.23, .49) | <.001 |
| CD-Short Version ^b^ | ODD/CD | ODD/CD+ADHD | -.11 (-.18, -.03) | .004 |
|  | ODD/CD | ADHD | .07 (.01, 13) | .028 |
|  | ODD/CD+ADHD | ADHD | .18 (.10, 25) | <.001 |
| CD-Full Item Pool ^b^ | ODD/CD | ODD/CD+ADHD | -.07 (-.12, -02) | .006 |
|  | ODD/CD | ADHD | .05 (.01, .09) | .016 |
|  | ODD/CD+ADHD | ADHD | .12 (.07, .17) | <.001 |
| Note. ^a^ comparisons based on Tukeys-HSD post-hoc test. ^b^ comparisons based on Games-Howell post-hoc test. ^c^ Group 1 - Group 2. | | | | |
